# The role of endotoxin in septic shock

**DOI:** 10.1186/s13054-023-04690-5

**Published:** 2023-10-19

**Authors:** John A. Kellum, Claudio Ronco

**Affiliations:** 1 Department of Critical Care Medicine, Center for Critical Care Nephrology, University of Pittsburgh, 600 Scaife Hall, 3550 Terrace Street, Pittsburgh, PA 15261 USA; 2 Spectral Medical Inc, Toronto, ON Canada; 3 International Renal Research Institute of Vicenza, IRRIV Foundation, Department of Nephrology, Dialysis and Transplantation, St. Bortolo Hospital, aULSS8 Berica, Via Rodolfi, 37, 36100 Vicenza, Italy; 4 Department of Medicine (DIMED), University of Padua, Via Giustiniani, 2, 35128 Padua, Italy

**Keywords:** Sepsis, Phenotypes, Septic shock, Endotoxin, Endotoxic septic shock

## Abstract

Septic shock can be caused by a variety of mechanisms including direct effects of bacterial toxins such as endotoxin. Annually, approximately 5–7 million patients worldwide develop sepsis with very high endotoxin activity in the blood and more than half die. The term endotoxic septic shock has been used for these patients but it is important to emphasize that endotoxin may be a factor in all forms of septic shock including non-bacterial etiologies like COVID-19 since translocation of bacterial products is a common feature of septic shock. A pattern of organ failure including hepatic dysfunction, acute kidney injury and various forms of endothelial dysfunction ranging from disseminated intravascular coagulation to thrombotic microangiopathy characterize endotoxic septic shock. However, while characteristic, the clinical phenotype is not unique to patients with high endotoxin, and the diagnosis relies on the measurement of endotoxin activity in addition to clinical assessment. Therapies for endotoxic septic shock are limited with immune modulating therapies under investigation and extracorporeal blood purification still controversial in many parts of the world.

## Introduction

Across the globe sepsis is now estimated to result in more than 11 million deaths a year [1] and septic shock, the most severe form, leaves nearly 40% of patients dead at hospital discharge [2]. Thus, even though sepsis care has improved, it remains a major problem around the world. Sepsis is also a heterogeneous and imprecise syndrome that likely includes multiple phenotypes, some of which may be amenable to specific therapies not included in routine sepsis bundles. Progress in developing new therapies for sepsis will almost certainly require focus on specific subsets of patients [3, 4], and no single therapy will be effective for all patients. Careful evaluation of patients for treatable diseases manifesting within the clinical classification of sepsis is important to improving care. Because sepsis is a common condition, it is easy to overlook unusual causes of organ failure and to succumb to confirmational bias about the nature of the patient’s illness [5]. Careful attention to past-medical and family history and selective use of an array of diagnostic testing and subspecialty input can help identify potentially treatable diseases masquerading as “typical” sepsis.

The pathophysiology of sepsis is complex with host susceptibility factors (age, environment, genetics, etc.) interacting with pathogen load, virulence, and various pathogen-associated molecular patterns (PAMPs) [6]. The best characterized PAMP is endotoxin and sepsis pre-clinical studies, including animal models, routinely use high-dose endotoxin. While endotoxin may seem less “fashionable” than it once was, PubMed citations continue to increase, surpassing 5000 per year in 2022. Endotoxin is a lipopolysaccharide component of the outer cell membrane of Gram-negative bacteria which can trigger a brisk host response and multiple types of acute organ failure. *Homo sapiens* are the most sensitive species to endotoxin in the animal kingdom even compared to other primates [7]. Rather than live bacteria, translocation of bacterial products from the gut is the dominant source of endotoxemia, and 70% of patients with septic shock and high endotoxin activity have negative blood cultures [8].

### Endotoxic septic shock?

Only about 10–15% of sepsis, or approximately one third to half of patients with septic shock, exhibit high levels of endotoxin activity in their blood [8]. The term endotoxic septic shock (ESS) has been used to define this subgroup of patients, and this group may benefit from anti-endotoxin therapy. However, while risk for ESS is greatest in patients with Gram-negative infection, sepsis secondary to Gram-positive organisms, fungi and some viruses (e.g., COVID-19 [9]) may also lead to ESS. Thus, we recommend that the term ESS be reserved for patients with proven endotoxemia (e.g., by endotoxin activity > 0.6 units) and not based on blood cultures or presumed source of infection. Worldwide about 5–7 million cases of ESS occur each year. ESS is particularly deadly. In an observational study, Adamik and colleagues reported a twofold increase in ICU mortality for patients with septic shock and high endotoxic activity, and these differences persisted for at least 90 days when mortality was < 50% with lower endotoxin activity and > 70% with ESS [10]. Interestingly, day 1 SOFA and APACHE II scores were identical between these patients.

### Endotoxin and pathophysiology and clinical manifestations of septic shock

**Fig. 1:**
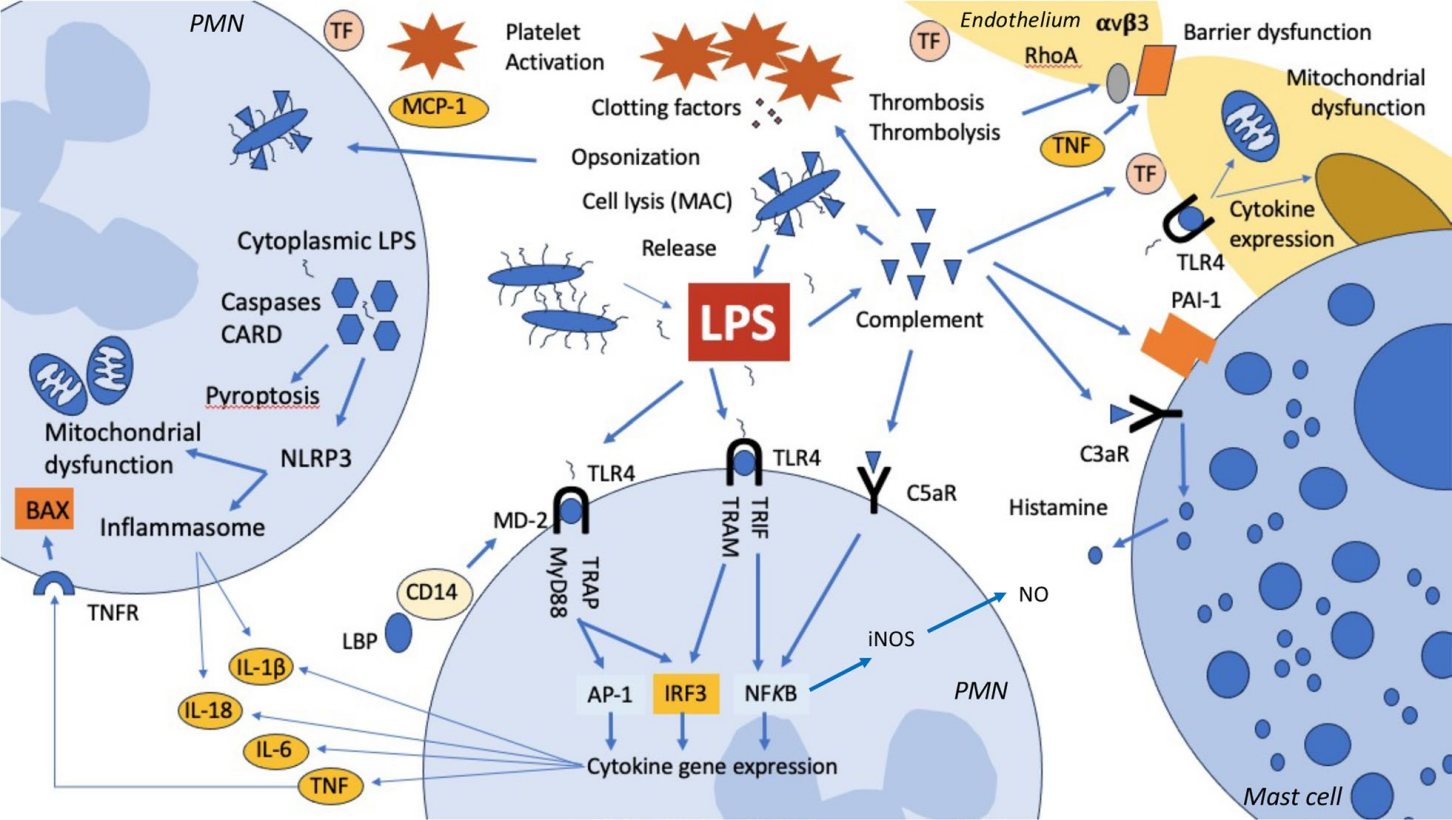
Mechanisms of endotoxic septic shock. Dominant mechanisms of LPS-induced cell damage. Endotoxin lipopolysaccharide (LPS) is released from Gram-negative bacteria in response to proliferation but greatly increased with bacterial cell death. TLR4/MD-2 (neutrophil in the lower field) is the primary receptor for extracellular LPS which engages multiple overlapping pathways leading to expression of cytokines and other inflammatory molecules. However, cytoplasmic LPS (left) is also sensed by caspase activation and recruitment domains and caspases 4 and 5 leading to NLRP3-mediated inflammasome activation. This process may also directly result in mitochondrial dysfunction as a TNF-BAX-mediated process shown in the lower left. LPS is also a potent activator of complement and C5a can directly induce NF*K*B-mediated inflammation. C3a signaling also leads to histamine release from mast cells (right). Complement activation can affect coagulation in numerous ways PAI-I and TF are induced, platelets become activated, and the clotting cascade is engaged. Fibrinogen fragments can induce endothelial barrier dysfunction mediated by alpha-v and beta 3 integrins in a RhoA-dependent fashion. αvβ3, alpha-v beta 3 integrin; AP-1, Activator protein 1 transcription factor; BAX, Bcl-associated X protein; CARD, caspase activation and recruitment domain; iNOS, inducible nitric oxide synthetase; IRF3, interferon regulatory factor 3; MAC, membrane attack complex; MCP-1, monocyte chemoattractant protein 1, MyD88, myeloid differentiation primary response 88; NF*K*B, nuclear factor kappa B; NLRP3, NLR family pyrin domain containing 3; NO, nitric oxide; PAI-1, plasminogen activator inhibitor-1; RhoA, Ras homolog gene family, member A; TF, tissue factor; TRAM/TRAP/TRIF, TLR adaptor molecules

**Fig. 2:**
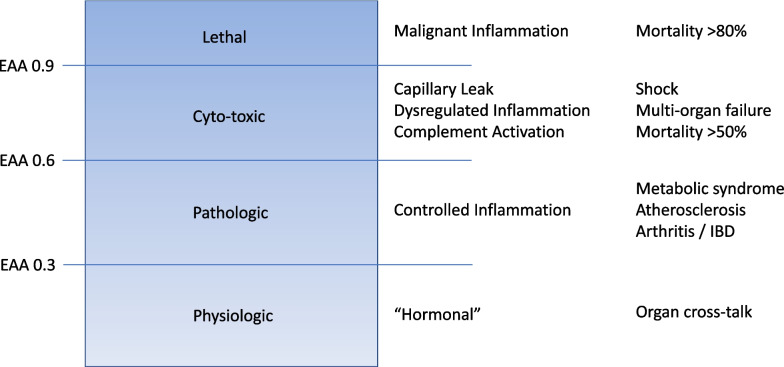
Relationship between endotoxin load and clinical manifestations. EAA, endotoxin activity assay

Endotoxin triggers inflammation through Toll-like receptor 4 (TLR4) in conjunction with myeloid differentiation factor 2 (MD-2) and cluster of differentiation 14 (CD14), the later also requiring lipopolysaccharide binding protein (LBP). In humans, the downstream signaling pathway from TLR4 activation involves three separate arms and is highly conserved across animal species. However, humans and other mammals detect endotoxin through multiple additional mechanisms (Fig. 1), including serum factors, intra- and extracellular proteins. The three major recognition mechanisms are (i) the TLR4-MD-2 receptor pathway, which detects extracellular endotoxin, (ii) the caspase 4/5 mechanism, which detects endotoxin in the cell cytoplasm (e.g., from intracellular bacterial infection), and (iii) complement which binds to endotoxin in the blood [7]. These various signaling pathways may help explain the diverse clinical manifestations of ESS. However, again it should be emphasized that endotoxin activity is on a continuum (Fig. 2) and virtually all patients with septic shock have some amount of endotoxin present. Even at low dose, endotoxin produces profound effects in humans across multiple organ systems [11]. Because endotoxin induces a brisk reaction in both complement and inflammation, high doses result in typical organ injury patterns which include shock, acute kidney injury (AKI), liver dysfunction, and endothelial injury with coagulation abnormalities/endothelial dysfunction. Akitomi and coworkers described whole blood gene expression profiling in a patient with ESS [12]. Comparative gene expression analysis of whole blood from the patient identified more than 2000 genes involving oxidative stress, neutrophil defensins, tumor necrosis factor-α/nuclear factor-κB, interleukin-8 and -6 signaling cascades, and pyruvate metabolism among others. In an unusual case of self-injection intravenously of high-dose endotoxin (1 mg), a patient developed profound shock, AKI, hepatic and endothelial dysfunction with relatively spared pulmonary, and neurologic function [13]. Accordingly, we recommend that term ESS be reserved for patients likely to benefit from anti-endotoxin therapy. Such patients are not only proven to have high concentrations of endotoxin, but also have a high burden of acute organ dysfunction.

### Diagnosing endotoxemia

Although this pattern of organ damage is characteristic, it is not specific to endotoxin. At least, 30 TLR4 ligands have been identified to date including multiple pathogen-associated molecular patterns (PAMPs) from bacteria but also viruses and fungi [14]. Furthermore, numerous endogenous ligands have been characterized most notably high-mobility group box 1 (HMGB1) protein and heat shock proteins (HSPs). Moreover, other members of the Toll-like receptor family can also recognize PAMPs and share many of the same downstream pathways. Thus, even careful clinical phenotyping cannot easy distinguish ESS from patients with septic shock but with lower levels of circulating endotoxin. This distinction is critical, however, because interventions targeting endotoxin will only be effective when high amounts of endotoxin are present. Rapid determination as to whether endotoxin or other inciting molecules are the primary drivers of septic shock is essential to providing precision medicine. Unfortunately, it is not easy to detect endotoxin in the bloodstream. Most endotoxin rapidly becomes sequestered by complement and molecules like LBP and HDL cholesterol such that “free endotoxin” is relatively scarce even when exposure is high. Still, the overall burden of endotoxin is related to survival. Endotoxin can be measured in whole blood using the endotoxin activity assay (EAA) and high endotoxin activity increases risk of death [10, 15]. EAA is an immunoassay that uses anti-lipid A monoclonal Ab and whole blood. Endotoxin in the blood sample binds with the Ab and this Ag–Ab complex stimulates neutrophils also in the sample. Reactive oxygen species produced by neutrophils are then measured by the luminol chemiluminescence reaction. Basal and maximally stimulated samples are measured in parallel as negative and positive controls, and endotoxin activity in the sample is expressed as a relative value (EAA level) [16]. A level of 0.60 or higher is considered the threshold for high endotoxin activity and is associated with increased ICU mortality [15]. Importantly, while EAA correlates with risk of death, there is still variation at the patient level with respect to the clinical response to endotoxin. Although humans are exquisitely sensitive to endotoxin, we have multiple defense mechanisms (e.g., complement, binding proteins) that can rapidly sequester an endotoxin challenge. Factors such as prior exposure, physiological reserve, and genetic variation, especially in genes controlling components of the complement system and leukocyte function [5], may influence the success of these defenses. Although ESS can be defined simply as the presence of high endotoxin activity in the setting of septic shock, patients with low organ failure burden (e.g., sequential organ failure assessment (SOFA) < 7) in this setting have low risk of death and do not appear to benefit from therapies targeting endotoxin [17]. In the EUPHRATES trial, 29-day mortality from ESS in patients with low organ failure was < 20% and was not affected by endotoxin removal [8].

### Phenotypic variation in septic shock

The reason that some patients with high levels of endotoxin have less severe manifestations while others have rapidly progressive organ failure and death is unclear. The level of endotoxin activity may be one explanation (Fig. 2). Approximately, 17% of patients in the EUPHRATES trial were found to have an EAA of 0.9 or greater [18]. This level is beyond the ability to accurately measure with EAA (equivalent to approximately > 4000 pg/ml of a standard endotoxin preparation of E. coli strain O111:B4) [19]. However, even for the same burden of endotoxin, patients can react differently. Because inflammation on a systemic level is dangerous, multiple endogenous regulatory mechanisms exist and are vital for survival. Both pro- and anti-inflammatory cytokines are released, and engagement of complement and coagulation cascades have built in “breaking mechanisms” ensuring the system is controlled as much as possible. Sepsis is the most common form of dysregulated inflammation, but others also exist. Syndromes such as cytokine expression in response to chimeric antigen receptor (CAR)-T therapy, macrophage activation syndrome (MAS), and atypical hemolytic-uremic syndrome (aHUS) are also examples of dysregulated inflammation.

Some of the phenotypic variation in ESS can be linked to genetic differences. A Danish study showed a near sixfold increase in the risk of death from infection before age 50 for adoptees whose biological parents also died from infection under age 50 [20]. However, despite great variation in host response, attempts to identify genetic variants that contribute to sepsis outcomes has proven challenging. Most genomic studies in sepsis have treated all patients as a single group, assuming shared genetic risk factors. They have also focused on correlations between common polymorphisms and sepsis outcome with limited functional studies to support associations [21, 22]. Recently, whole exome sequencing (WES) has become more affordable, and studies have been undertaken in sepsis [23]. One such study hypothesized that variation in certain genes implicated in the pathogenesis of syndromes such as MAS and aHUS would be more common in patients with sepsis manifesting extreme inflammation. Using serum ferritin > 7000 ng/ml as a screen, investigators performed WES on six patients. All six exhibited one or more gene variants associated with hyperinflammation and five out of six had variants associated with MAS and/or aHUS. While all the variants associated with MAS and aHUS reported in this study have been classified as pathogenic or likely pathogenic, they may or may not have been causal. Moreover, even if genetic variation played a role in the extreme phenotypes exhibited in these cases, the application of immunomodulatory therapies to septic individuals with these variants is of unclear benefit or harm. However, these findings provide evidence that screening select sepsis patients can identify unappreciated heritable disease and could facilitate a genome-driven precision medicine.

Importantly distinct clinical subtypes that resemble MAS and aHUS can be found when large datasets of patients with sepsis are examined. Seymour and colleagues [3] used machine learning to derive clusters of clinical characteristics (i.e., phenotypes) from patients meeting the Sepsis-3 criteria [24] within 6 h of hospital presentation. K-means clustering was applied to all clinical and laboratory variables in the electronic health record (29 in all) from 16,552 patients and then validated in a second database (n = 31,160) and in prospective cohorts from observational studies and RCTs (n = 5320). Optimal fit was obtained with four derived phenotypes (α, β, γ, and δ) and host response biomarkers (e.g., cytokines); organ failure patterns and survival varied considerably across phenotypes. Interestingly, while all phenotypes included some dysfunction across organs, those associated with MAS and aHUS (i.e., kidney, liver and coagulation abnormalities) tended to cluster in one phenotype (phenotype δ). The δ-phenotype was present in 10–15% of patients across datasets and was associated with a dramatically higher mortality rate (32% in-hospital mortality compared to 2% for the α-phenotype). It is logical to posit that endotoxemia may be a driver of this phenotype in many patients. Since endotoxin is not routinely quantified and since MAS and aHUS are often missed, further research is needed to confirm or refute the hypothesis that these conditions are driving the δ-phenotype.

### Treatment for endotoxic septic shock

For patients with MAS and aHUS, immune modulating therapies are now available [5]. However, many patients may exhibit incomplete manifestations of these conditions and the diagnosis may be unclear. Furthermore, treatment of patients with sepsis using drugs that target cytokines, (e.g., anakinra, an interleukin 1 receptor antagonist) or complement (e.g., eculizumab, monoclonal antibody to C5) could be harmful if infection is still active.

Rather than treating the syndrome with immune modulating therapies, another approach would be to target endotoxin directly. However, multiple efforts to block endotoxin signaling have failed in clinical trials despite encouraging pre-clinical data. Indeed, efforts to neutralize endotoxin began in the 1970s and accelerated as the molecular structure of endotoxin was characterized [25]. Various antibodies to endotoxin have been studied but clinical trials testing these therapies have been discouraging. However, few studies have examined the effect of these treatments in patients with detectable endotoxemia [26, 27]. An analysis of HA-1A found that this monoclonal antibody reduced mortality for 27 patients with endotoxin in their blood but not for 55 patients without detectable endotoxin [27]. In general though, results in the endotoxin positive sub-groups of patients have not been positive [28]. The reasons for the disconnection between strong pre-clinical data, biologic rationale and negative trials have been pondered in multiple reviews [25]. Potential explanations include problems with the agents themselves, study populations, and timing of therapy.

An alternative strategy to pharmacologic neutralization of endotoxin is removal of the molecule using extracorporeal therapy. Several methods have been tried but the most prevalent is polymyxin B hemoadsorption (PMX). Polymyxins are a group of cyclic cationic polypeptide antibiotics which have well-characterized endotoxin binding. While toxicity limits the clinical use of polymyxin B as an antibiotic, the compound can be bound to a hemoadsorption column and circulating endotoxin can be effectively removed through exposure to immobilized polymyxin B without the systemic toxicity. This method has been available in Japan since 1994 and received CE mark approval in Europe in 1998. More than 100,000 patients have been treated in more than a dozen countries [29]. Analyses of clinical data from a national Japanese database using propensity matching and other techniques have demonstrated benefit in the range of 3–7% absolute risk reduction for hospital mortality [17, 30]. No clinical trials have been adequately powered to find an affect size in this range. Of the three largest trials to date, only the EUPHAS trial found a significant improvement in survival [31]. The primary endpoint for EUPHAS was reversal of shock over 72h, and this was significantly improved with PMX P < 0.001. However, a secondary endpoint, 28-day mortality, was 32% in patients treated with PMX and 53% with conventional therapy (hazard ratio: 0.43; 95% confidence interval: 0.20–0.94). The ABDOMIX trial in France [32] was negative but the study enrolled a much lower risk population (control group mortality < 20%) and a median SOFA score of 10. Furthermore, when endotoxin mass was measured after the completion of the trial, mean values were quite low [33] compared to prior studies in sepsis [34].

The EUPHRATES trial in the US [8], the largest trial to date, did not find a survival benefit for PMX. However, the EUPHRATES trial was significantly different in design to other trials. Midway through the trial, enrollment was restricted to patients with Multiple Organ Dysfunction Score (MODS) of 9 or less [35], and the group with MODS > 9 became the primary analysis cohort. This change was prompted by evidence that any benefit appeared to be limited to patients with greater organ dysfunction. A similar conclusion was recently reached by Fujimori et al. in analysis of more than 4000 patients from Japan [17]. In this analysis, the therapy was most effective for patients with more organ failure.

Another significant difference between the EUPHRATES trial and other studies however, was the use of the EAA test and enrollment into the EUPHRATES trial was restricted to patients with septic shock who were found to have EAA 0.60 or higher. Overall, the EUPHRATES trial showed that even in the per protocol analysis restricted to patients with a MODS > 9, 28-day mortality was 33% with hemoperfusion versus 36.4% with sham, a difference that was not statistically significant [8]. However, the EAA assay cannot precisely quantify circulating endotoxin when EAA levels are 0.90 or greater and values in this range may not represent treatable levels. A reanalysis of the EUPHRATES trial data revealed that when patients with EAA at or above 0.9 are removed, 28-day mortality was 26.1% for polymyxin B hemoperfusion versus 36.8% for sham (risk difference 10.7%, OR 0.52, 95% CI (0.27, 0.99), *P* = 0.047) [36]. These findings prompted the design of an ongoing trial in the US (NCT03901807).

Finally, even for patients with high levels of endotoxin management of sepsis will always necessitate a range of therapies depending on the nature and severity of organ dysfunction. Antibiotics and source control along with supportive therapy remains vital. AKI complicates the majority of these cases, and many will require renal replacement therapy [37]. Blood purification strategies targeting multiple aspects of the ESS syndrome may also be considered [38] especially given that 28-day mortality may still be > 30% even when endotoxin removal is applied. Such strategies may target downstream mediators using broad-spectrum sorbents (e.g., Cytosorb, HA380). Future trials will be needed to establish the effectiveness of this approach.

## Conclusions

Endotoxic septic shock (ESS), defined by high endotoxin activity (e.g., EAA > 0.6) and organ failure (e.g., SOFA > 7, appears to be a subtype of sepsis accounting for approximately 5–7 million cases annually worldwide. Some patients develop severe hyperinflammation, hepatic dysfunction and disseminated intravascular coagulation resembling macrophage activation syndrome (MAS), and others resemble atypical hemolytic-uremic syndrome (aHUS); some patients have features of both. These subtypes are characteristic of the sepsis δ-phenotype and may have a genetic predisposition. ESS has a mortality in excess of 50%, and therapies are limited. Efforts to apply immune modulating therapies to ESS are under investigation, as are studies to expand the use of extracorporeal endotoxin removal as well as other forms of blood purification.

## Data Availability

Not applicable.
